# The Absolute Threshold of Colour Vision in the Horse

**DOI:** 10.1371/journal.pone.0003711

**Published:** 2008-11-12

**Authors:** Lina S. V. Roth, Anna Balkenius, Almut Kelber

**Affiliations:** 1 Department of Cell and Organism Biology, Vision Group, Lund University, Lund, Sweden; 2 Department of Plant Protection Biology, The Swedish University of Agricultural Sciences, Alnarp, Sweden; Max-Planck-Institut fuer Neurobiologie, Germany

## Abstract

Arrhythmic mammals are active both during day and night if they are allowed. The arrhythmic horses are in possession of one of the largest terrestrial animal eyes and the purpose of this study is to reveal whether their eye is sensitive enough to see colours at night. During the day horses are known to have dichromatic colour vision. To disclose whether they can discriminate colours in dim light a behavioural dual choice experiment was performed. We started the training and testing at daylight intensities and the horses continued to choose correctly at a high frequency down to light intensities corresponding to moonlight. One Shetland pony mare, was able to discriminate colours at 0.08 cd/m^2^, while a half blood gelding, still discriminated colours at 0.02 cd/m^2^. For comparison, the colour vision limit for several human subjects tested in the very same experiment was also 0.02 cd/m^2^. Hence, the threshold of colour vision for the horse that performed best was similar to that of the humans. The behavioural results are in line with calculations of the sensitivity of cone vision where the horse eye and human eye again are similar. The advantage of the large eye of the horse lies not in colour vision at night, but probably instead in achromatic tasks where presumably signal summation enhances sensitivity.

## Introduction

Between a sunny summer day and a moonless night there is a 1000 million-times intensity difference [1]. To function over this huge range of intensities puts an eye at very high demands. Therefore, most vertebrates have duplex retinae with multiple types of cones that can contribute to colour vision in daylight intensities and very light-sensitive rods for vision in dim light [2].

At night when light is dim it is vital for the eye to capture as many photons as possible to allow for a strong visual signal. Common optical adaptations in animals active at night are consequently large eyes with large pupils and short focal lengths (thus, a low f-number) to concentrate the sparse photons onto fewer photoreceptors. In addition, the visual signals from neighbouring photoreceptors can be summed in space and in time to generate a higher signal-to-noise ratio at night at the expense of spatial and temporal resolution [1], [3].

Colour vision is compromising sensitivity since the colour signal requires a comparison between signals from photoreceptor types with different spectral sensitivities. For vertebrates this usually means a comparison between at least two different types of cones [4], [5], even though rods influence colour perception at low light intensities. As the signals from the cones are compared to generate a colour signal, the photoreceptor noise adds up and becomes relatively larger generating a low signal-to-noise ratio.

**Figure 1:**
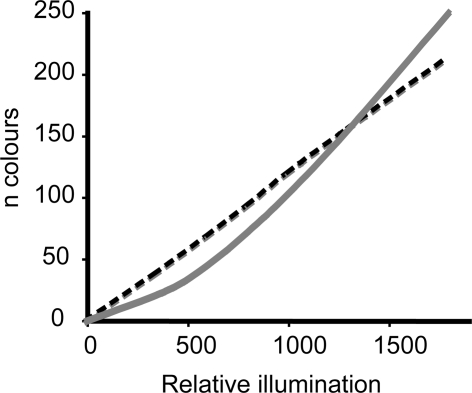
The relative number of colours (n colours) that a dichromat (dashed black line) and a trichromat (grey solid line) can discriminate in dim light. Due to the high noise levels in dim light intensities a dichromat should theoretically discriminate more colours than a trichromat. Adapted from [6].

Spectral pooling–summation of signals from all photoreceptors independently of their maximum spectral sensitivity–could be one way of strengthening the signal. However, this means sacrificing colour vision. In dim light a monochromat should, due to lower noise levels, be able to discriminate more shades than a dichromat [6]. For the same reason, dichromats, comparing signals between only two cone types, fare better than trichromats [6]. Trichromatic colour vision, such in humans, demands two comparisons of signals from three cone types thus generating a low signal-to-noise ratio. Therefore, dichromatic vision theoretically generates a stronger signal-to-noise ratio and allows discrimination of colours at dimmer light conditions compared to trichromatic vision (Fig. 1).

It was earlier thought that animals sacrifice colour vision in favour of absolute sensitivity at night. However, colour information is rich and valuable also during the evening and night. When the light intensity and the colour of light change during dusk and dawn, colour information is more reliable than brightness and obviously there are animals taking advantage of this [7]. During the last decade nocturnal hawkmoths and geckos were shown to be able to discriminate colours in dim light [8], [9]. Nocturnal geckos, which only have cones in their retinae, have adapted their eye and cones to become sensitive enough to make use of colour information at night.

How about vertebrates that possess both rods and cones? To answer this question, we studied colour vision in an arrhythmic mammal, the horse. The eyes of horses are among the largest terrestrial eyes and their pupil is very mobile and can dilate greatly to catch the sparse photons at night [2], [10]. In addition, the reflecting tapetum lucidum in the back of the horse eye, gives the non-absorbed photons a second chance to be captured by the photoreceptors and thereby enhances sensitivity further [11].

Grzimek [12] showed behaviourally that horses can see colours during the day, and there have been several studies confirming these results [13]–[16]. The aim of our study was to behaviourally determine the absolute threshold of colour vision in horses, and to compare it to the colour vision threshold of humans. For this purpose we also collected information on the photoreceptors and the optical properties of the eye and determined the optical sensitivity of the horse eye. This allows us to compare the colour vision thresholds both with regards to absolute light intensity and to the optical sensitivity of the eyes.

## Results

**Figure 2:**
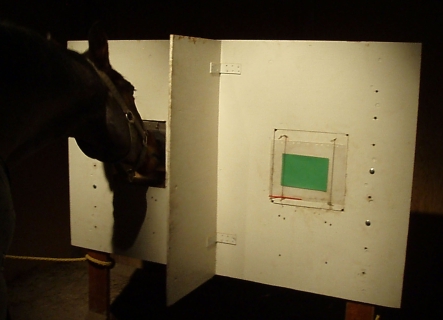
The dual choice apparatus wall. The door presenting the rewarding stimuli is unlocked and enables the horse to reach the pieces of carrots behind the door.

**Figure 3:**
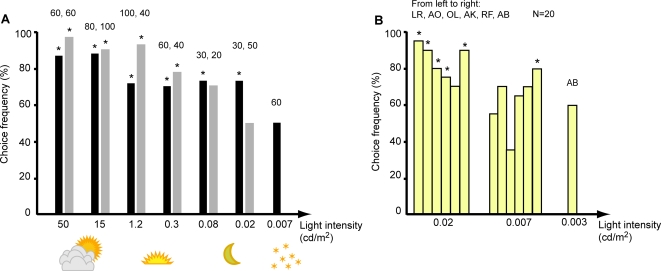
The choice frequencies at different light intensities for two horses (A) and six human subjects (B) as a comparison (binominal tests *P<0.05). The data presented for Chap (black bars) and Rosett (grey bars) start from the second day they performed at least 70% correct choices and the number of choices are written above each bar. Chap discriminated colours at 0.02 cd/m^2^ but chose randomly at 0.007 cd/m^2^. Six human subjects (B) were presented with 20 combinations per intensity. Initials of the human subjects are listed from left to right as they appear in the graph. All but RF chose the positive stimulus significantly more often at 0.02 cd/m^2^. At 0.007 cd/m^2^ all but AB failed to discriminate the colours. AB did not fail until the light intensity was lowered to 0.003 cd/m^2^.

We trained three horses in a dual choice experiment with one positive rewarded stimulus and one negative unrewarded stimulus (Fig. 2). Chap and Rosett were trained to blue as the positive stimulus and Rex was trained to green as positive stimulus. When the horses had reached a specified learning criterion of at least 70% correct choices for a number of days, the light intensity was lowered. All three horses chose correctly at significantly high frequencies, for all intensities higher than or equal to 1.2 cd/m^2^ (Fig. 3a), which is comparable to light intensities at sunset. At lower light intensities, Rex totally lost the motivation for the experiment, and after that chose randomly even in tests in brighter light (15 cd/m^2^). He was therefore excluded from further testing and his data are not presented in the result figure. Rosett lost interest in comparing the two stimuli at a light intensity of 0.02 cd/m^2^ and developed a preference for the right side. After this point, she preferred the right side and performed poorly, even when the light intensity was increased to the highest intensity used in this experiment. The last three days she chose the left side in only 6 out of 30 choices. After this point she was excluded from further testing.

Chap still chose the positive blue stimulus in a total 31 out of 40 presentations, at 0.02 cd/m^2^ (binominal tests, P<0.05, note that only the last 30 choices are presented in Figure 3). However, at the next lower intensity, 0.007 cd/m^2^, he failed to choose correctly and made only 30 correct choices in 60 presentations. Directly after the last day of tests in the dimmest light intensity, Chap was tested in bright light again (15 cd/m^2^) to determine whether he had lost motivation. In this control, he confidently chose the positive blue stimulus nine out of ten times (P<0.05), ensuring that his motivation still was high. Chap's results thus suggest that at least some horses can discriminate colours at a light intensity similar to moonlight intensities (0.02 cd/m^2^).

The results in Figure 3a include choices starting with the second day the horses reached the learning criterion of at least 70% correct choices. All choices were examined both with respect to calculated quantum catches for the long wavelength-sensitive cone and the rods (see methods) to assure that no brightness cues had guided the horse in its choices. Of Chap's correct choices, 52% were made when the positive stimulus was darker than the negative stimulus with regard to the quantum catches in the long-wavelength-sensitive cone. The same number was found for the rods. Thus, Chap did not use brightness in his choices.

Six human subjects were tested with the same stimuli and experimental apparatus as a comparison to the horse result. Only twenty presentations at the intensities around threshold were performed. At 0.02 cd/m^2^, the results varied from 70–95% correct choices and choices by all but one subject (RF, 70%) were significantly different from chance (binominal tests, P<0.05; Fig. 3b). At 0.007 cd/m^2^ the choice frequency varied between 35 and 70% for five of the six human subjects. Thus, all five subjects failed to discriminate the colours at this dim light intensity. However, the sixth human subject (AB) chose correctly in 80% of the twenty choices (P<0.05) at 0.007 cd/m^2^ and did not fail to choose correctly until the light intensity was lowered to 0.003 cd/m^2^.

### Optical sensitivity

**Figure 4:**
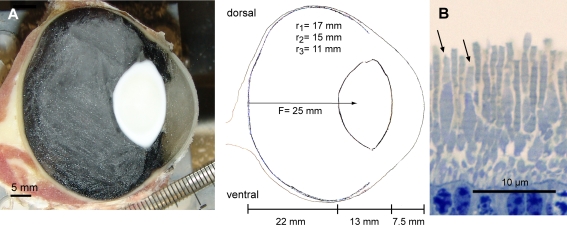
The distance from retina to the nodal point was 25 mm in the frozen, sagitally sectioned horse eye (A). The outer segment of the bulkier cones (B) has a diameter of 1 μm and a length of 4 μm (arrows).

Horse eye dimensions were obtained by sections through freshly frozen eyes (Fig. 4a). Assuming a well-focused image on the retina, a posterior nodal distance (here also called focal length) of 25 mm was found for the horse (Fig. 4a). The cones of a different horse eye were examined under the light microscope. They measured 1 μm in diameter and the outer segments were 4 μm long (Fig 4b), which is similar to the cone dimensions measured in the bovine retina [17].

The measurements taken from the horse eyes are summarized in Table 1 together with the human data that were obtained from the literature [18]–[20]. The optical sensitivity that we determined for cone vision of horses and humans corresponds to the ability of a cone to capture photons from an extended source of white light through the opened pupil in light intensities corresponding to moonlight (See methods section, Eqn. 2). This equals the sensitivity of the eyes without any signal summation, at the lowest light intensity at which colour discrimination was possible in our experiments.

**Table 1:** The optical sensitivity for the cones of the human eye and the horse eye at intensities corresponding to moonlight.

	Symbols	Human (*Homo sapiens*)	Horse (*Equus caballus*)
Absorption coefficient	k	A 0.035	A 0.035
Pupil diameter	A (μm)	B 7000	30000
Focal length	f (μm)	A 16700	25000
Cone diameter	d (μm)	B 1.5	1
Length of cone outer segment	l (μm)	C 30	(4)
Double length with tapetum	l (μm)		D 8
Optical sensitivity for white light	S_w_(μm^2^ sr)	0.08	D 0.1

A [18]

B [19] An average of the cone diameter in parafovea and perifovea is used.

C [20]

D The tapetum is the reason for the double effective length of the cone outer segments.

Even though the horse eye is very large and has a relatively short focal length the calculated sensitivity for cone vision is similar to that of humans, due to the short outer cone segments in the horse retina. Using obtained data and Eqn. 2, the eye of the horse yields a sensitivity of 0.1 μm^2^sr when the reflecting tapetum lucidum in the back of the horse retina is taken into account. Due to the tapetum the non-absorbed photons have a second chance to be absorbed by the outer segment of the photoreceptors and therefore it doubles the effective length of the outer segment. The human eye yields a sensitivity of approximately 0.08 μm^2^sr (Table 1). The sensitivity of horse cone vision is thus very similar to that of humans. As a comparison, an animal truly adapted to dim light vision, the toad (*Bufo*) has a calculated optical sensitivity of 2.41 μm^2^sr [18]. The optical sensitivity of human rod vision is 0.085 μm^2^sr and that of horses is 0.13 μm^2^sr when no signal summation is taken into account (data not shown).

## Discussion

The large eye, and the fact that dichromatic colour vision is superior to trichromatic vision in very dim light [6] led to our initial hypothesis that horses might discern colours at lower light intensities than humans. In behavioural experiments with dogs, Rosengren [21] found that one of her cocker spaniels could discriminate colours at a somewhat lower light intensity than humans. Unfortunately no light intensity measurements were performed during her experiment and “deepening twilight” is the only description of the light level in her experiment.

In our experiment the motivation of some horses dropped in low light intensity and the horses were easily distracted. It is possible that more motivated horses are able to discriminate colours at somewhat lower light intensities. However, one horse was motivated throughout the experiments. Our study suggests that horses and humans have similar thresholds of colour vision and discriminate colours in moonlight intensities (0.02 cd/m^2^). Still, colour seems to be a less salient stimulus for horses than for humans since only one of the three horses in our study performed well at 0.02 cd/m^2^. We also found some variation in the threshold of human colour vision. The experimenter (AB), who managed to discriminate colours even at an intensity of 0.007 cd/m^2^, seemed to have coarser spatial resolution at low light intensities than the other subjects since she needed help to write down her choices. These individual differences among humans may deserve further attention and studies.

The low light intensities that we used in the behavioural experiment are likely to be mesopic to horses. For humans, this means that colours appear less and less saturated, which indicates that rod signals become stronger while cone signals become weaker. A similar process could influence horse colour vision even more than it does for us since horses do not have a rod-free area in the retina as we do in the centre of our fovea. On the contrary, rods dominate most of the horse visual streak and with a smaller pupil, the rod signals would probably take over at even higher light intensity than in humans.

The large eye of the horse is not adapted to nocturnal colour vision. Instead it probably favours achromatic rod vision in dim light where the large eyes and pupils are exceptionally good at catching light and where signal summation presumably enhances sensitivity without too much loss in spatial resolution. With the now confirmed colour vision ability in moonlight intensities, horse colour vision still functions over the largest changes in illumination colour that occur during the sunset and twilight period [7]. At dimmer light intensities the ability to detect movements from possible predators may be the most important visual task, and achromatic vision may therefore be favoured.

## Methods

### Animals

We trained three horses with normal vision in a behavioural experiment; Chap, a 14 year old half blood gelding, Rex, a 11 year old thoroughbred gelding and Rosett, a Shetland pony mare of an honourable age of 33. Eyes from a one-year-old half blood mare and a twelve-year-old draft mare, obtained directly after their death from a veterinary hospital in Helsingborg, Sweden, were used to obtain measurements for the calculation of the sensitivity of the eye. Both mares were put down after decision of their owners only, and even though the horses were unwell their eyes were still fine and valuable for our study. All experiments were approved by the Swedish animal welfare agency (M148-05) and the Swedish board of Agriculture (83-5942/08).

### Stimuli and apparatus

The horses were trained to discriminate between blue and green stimuli. To make intensity an unreliable cue five different brightness versions of blue and seven different brightness versions of green were used as stimuli. All stimulus colours were printed on a printer (Canon Pro 9000) on white Munken Pure Copy paper (Artic Paper Munkedals AB, Munkedal, Sweden) in the size 15×21 cm. The stimuli were kept in transparent plastic cases that did not influence the reflectance of the stimuli but kept them clean. The reflectance *S*(λ) of all stimuli and the spectrum of the illumination *I*(λ) used in experiment (halogen spotlights) were measured using a spectroradiometer (S2000, Ocean Optics Inc., Dunedin, FL, USA). The spectral sensitivity *R*_i_(λ) of photoreceptor *i* was calculated from the horse cone sensitivity peaks (428 and 539 nm) [22] and a typical mammal rod peak sensitivity (500 nm) [23] using the Stavenga-Smits-Hoenders rhodopsin template [24]. The relative number of quanta Q_i_ absorbed by the horse's cone type *i* looking at a stimulus was calculated using Equation 1.
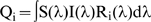
(1)


Both the quantum catch for the long wavelength-sensitive cone and the quantum catch for the rods (Table 2) were taken into consideration when combining the different “brightness versions” of blue and green for the training sessions. An equal number of times, the positive stimulus was brighter or darker than the negative stimulus and the stimulus pairs were presented in a pseudo-random order. The results were examined to make sure there was no correlation between stimulus brightness and choice frequency, i.e. horses did not choose the brighter or darker stimulus but the correct colour.

**Table 2:** Relative quantum catches for horse photoreceptors

Colours	Q _SWS_	Q _LWS_	Q _Rod_	Colours	Q _SWS_	Q _LWS_	Q _Rod_
Green 1	99	857	543	Blue 1	151	622	475
Green 2	38	747	411	Blue 2	131	463	382
Green 3	57	683	434	Blue 3	119	378	318
Green 4	34	472	315	Blue 4	106	322	267
Green 5	27	582	333	Blue 5	80	235	197
Green 6	17	339	219				
Green 7	15	306	202				

Seven shades of green and five shades of blue were tested and the relative quantum catches for the long wavelength-sensitive (LWS) cone type and rods were calculated according to Eqn. 1. These values were used to make sure that brightness of the colour stimuli were comparable for the horse. Relative quantum catches of the short wavelength-sensitive (SWS) cone type is included for comparison.

The dual choice apparatus (Fig. 2) was described in Roth et al. [16] and was similar to the one used by Macuda and Timney [15] and Geisbauer et al. [13]. In short, the apparatus consisted of a light grey wall with two lockable doors on either side of a divider, to force the horse to make a choice. Stimuli were presented on both doors. The door with the negative stimulus was locked and the door presenting the positive stimulus was unlocked such that the horse could reach pieces of carrots as reward.

### Behavioural experimental procedure

The experiment took place in a windowless barn between October 2007 and January 2008. Horses were trained in several steps to associate a colour with a reward of carrots. The horses were first made familiar with the experimental apparatus by leading them to the open apparatus doors by the halter, rewarding them with carrots. In the next step, they were taught to open the doors and reach for the carrot pieces by themselves. At this stage both doors showed positive stimuli (blue for Chap and Rosett, and green for Rex).

Depending on the performance of the horses it took 2 to 4 days before we started to release the horses three meters in front of the apparatus and introduced them to the training sessions with both negative and positive stimuli. Normally they were presented with 20 combinations each day, five days a week. However, at the three lowest light intensities, we allowed the horses to dark-adapt for five to fifteen minutes prior to testing and then, to avoid loss of motivation, we only presented ten stimuli per day.

For initial training we used the fluorescent tube that was present in the barn (50 cd/m^2^) as illumination source. A learning criterion was set to 75% correct choices for 20 combinations, which is significant according to the binominal test (P<0.05). When the horses had reached the learning criterion during three days, we lowered the light intensity and started using two halogen spot lights (15 cd/m^2^). Lower light intensities were achieved by adding neutral density filters (0.6 Neutral density filters, 210 Lee filters) in front of the halogen spots, and the achieved intensities were measured using a radiometer (International light IL 1700).

When the horses reached the learning criterion at one intensity level for a number of days we dimmed the light the following day. When a horse was tested at 0.08 cd/m^2^, at least five minutes were allowed for dark adaptation before testing started. When we tested the horses at 0.02 cd/m^2^ or lower light levels, we allowed them to dark-adapt five minutes at 0.08 cd/m^2^. The horses were then presented with five stimulus combinations to test adaptation before we lowered the intensity further to 0.02 cd/m^2^ and waited five more minutes. By then even the human experimenters were well dark-adapted and the horses were presented ten stimulus combinations during the session. The learning criterion was 70% during three days when presenting only ten combinations.

Six human subjects (including the authors) were asked, and gave their written consent, to perform the very same experiment; three women, which included both the experimenters, and three males. Experiments were conducted according to Swedish ethical regulations and due to the uncomplicated nature of the experiments no special permission was required. The human subjects were tested with 20 stimulus combinations at two light levels, 0.02 cd/m^2^ and 0.007 cd/m^2^. One of the female experimenters (AB) was also tested at 0.003 cd/m^2^.

### Optical sensitivity of the eye

The eyes of one twelve years old mare were frozen and sectioned sagitally in a cryostat. Pictures were taken every 0.5 mm to obtain the largest eye dimensions (Fig. 4a). For calculations of the posterior nodal distance (from nodal point to retina, here also called focal length), we applied the Gullstrand model (for calculations see [2]). As refractive index of the aqueous humour and the vitreous humour a value of 1.335 was used, which is similar to values previously used for horses and other mammals [25], [26]. Since the horse has a lens with different concentric zones of different refractive indices [27] the effective lens index will be higher if treated as one homogenous lens. We assume that the optical system of the horse is well focused. Therefore, to achieve a calculated focused image on the retina a total refractive index of 1.507 was used for the lens, which is close to the index used in Nicolas' study (1.49) [28] but much higher than the index used in Sivak and Allen's study (1.43) [26]. In our study, assuming a well-focused image on the retina, a posterior nodal distance of 25 mm for the horse was found (Fig. 4a).

Pieces of retina from a one year old half blood mare were fixated 2% paraformaldehyde, 2% glutaraldehyde with cacodylate buffer over night followed by post fixation in 1% osmium tetroxid, dehydration and embedding in Epon. Semithin sections dyed with methylene blue were used for light microscopy. From the histological sections we obtained the diameter *d* and the length *l* of the cone outer segments. The pupil diameter *A* of the horse was determined from photographs of living horses at a light intensity corresponding to moonlight.

To calculate the optical sensitivity *S_w_* for white light for horses we used the obtained measurements on pupil size, focal length (posterior nodal distance) and cone dimensions (Eqn. 2) [18], [20]. The opical sensitivity *S_w_* gives the number of photons absorbed by a photoreceptor when looking at an extended source of white light (Eqn 2):
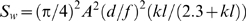
(2)where *k* is the absorption coefficient of the receptor, and *A* is the pupil diameter. A typical vertebrate photoreceptor absorption coefficient *k* of 0.035 was taken from Warrant and Nilsson [18].
